# A Study of Hyaluronic Acid’s Theoretical Reactivity and of Magnetic Nanoparticles Capped with Hyaluronic Acid

**DOI:** 10.3390/ma17061229

**Published:** 2024-03-07

**Authors:** Mihaela Răcuciu, Simona Oancea, Lucian Barbu-Tudoran, Olga Drăghici, Anda Agavriloaei, Dorina Creangă

**Affiliations:** 1 Environmental Sciences and Physics Department, Faculty of Sciences, Lucian Blaga University of Sibiu, Dr. I. Ratiu Str., no. 5–7, 550012 Sibiu, Romania; 2 Agricultural Sciences and Food Engineering Department, Lucian Blaga University of Sibiu, Dr. I. Ratiu Str., no. 7–9, 550012 Sibiu, Romania; simona.oancea@ulbsibiu.ro (S.O.);; 3 Electron Microscopy Integrated Laboratory, National Institute for R&D of Isotopic and Molecular Technologies, Donat Str., no. 67-103, 400293 Cluj-Napoca, Romania; lucian.barbu@itim-cj.ro; 4 Electron Microscopy Laboratory “Prof. C. Craciun”, Faculty of Biology and Geology, Babes-Bolyai University, Clinicilor Str., no. 5–7, 400006 Cluj-Napoca, Romania; 5 Biophysics and Medical Physics Laboratory, Faculty of Physics, “Alexandru Ioan Cuza” University, 11, Carol I Blvd., 700506 Iasi, Romania; anda.agavriloaei@yahoo.com (A.A.); mdor@uaic.ro (D.C.)

**Keywords:** magnetic nanoparticles, hyaluronic acid, iron oxide, nanoparticles’ size, biomedical uses

## Abstract

Hyaluronic acid (HA) has attracted much attention in tumor-targeted drug delivery due to its ability to specifically bind to the CD44 cellular receptor, which is widely expressed on cancer cells. We present HA-capped magnetic nanoparticles (HA-MNPs) obtained via the co-precipitation method, followed by the electrostatic adsorption of HA onto the nanoparticles’ surfaces. A theoretical study carried out with the PM3 method evidenced a dipole moment of 3.34 D and negatively charged atom groups able to participate in interactions with nanoparticle surface cations and surrounding water molecules. The ATR-FTIR spectrum evidenced the hyaluronic acid binding to the surface of the ferrophase, ensuring colloidal stability in the water dispersion. To verify the success of the synthesis and stabilization, HA-MNPs were also characterized using other investigation techniques: TEM, EDS, XRD, DSC, TG, NTA, and VSM. The results showed that the HA-MNPs had a mean physical size of 9.05 nm (TEM investigation), a crystallite dimension of about 8.35 nm (XRD investigation), and a magnetic core diameter of about 8.31 nm (VSM investigation). The HA-MNPs exhibited superparamagnetic behavior, with the magnetization curve showing saturation at a high magnetic field and a very small coercive field, corresponding to the net dominance of single-domain magnetic nanoparticles that were not aggregated with reversible magnetizability. These features satisfy the requirement for magnetic nanoparticles with a small size and good dispersibility for long-term stability. We performed some preliminary tests regarding the nanotoxicity in the environment, and some chromosomal aberrations were found to be induced in corn root meristems, especially in the anaphase and metaphase of mitotic cells. Due to their properties, HA-MNPs also seem to be suitable for use in the biomedical field.

## 1. Introduction

Nowadays, magnetic nanoparticles seem to represent one of the most important classes of nanomaterials due to their diverse applications in the biomedical, industrial, and environmental sectors.

The biomedical uses of magnetic nanoparticles are related to both in vitro applications (separation and purification [1,2] and magnetic solid-phase extraction [3]) and in vivo procedures, including in human body investigation and treatment through nuclear magnetic resonance imaging [4,5] and in tumor hyperthermia through magnetically assisted and magnetically targeted drug delivery [6,7,8].

The most interesting industrial uses in recent years are thought to be related to the MNPs’ remarkable potential for the petroleum industry [9], since their large area to volume ratio, magnetic features, and surface interaction properties make them suitable for various applications in targeted adsorption, guided transportation, and local heating.

As for environmental science applications, the adsorption properties of the MNP surface suggest their suitability for air purification, especially given the issue of global warming, which has increased researchers’ attention towards new procedures for CO_2_ adsorption [10] on membranes incorporating MNPs.

Regarding the biomedical applications of magnetic nanoparticles, over the last few decades, an increasing number of scientists have been attempting to find additional methods to improve the efficacy of the usual methods of cancer treatment, like chemotherapy, surgery, radiotherapy, and so on. Releasing anticancer drugs precisely to the tumor site can improve the efficacy and reduce the side effects of treatment on the patient’s body [11]. With its continuous development, nanotechnology could be one of the best options to enhance the success rates of oncologic chemotherapy, with well-targeted drug nanosystems able to be delivered through the blood–tumor barrier [12]. Therefore, in recent years, drug delivery nanotechnology has been extensively studied to improve the interactions of active compounds in target areas by controlling the distribution of the chemotherapeutic drug. Recently, magnetic nanoparticles have been used in drug delivery systems from the outside for drug-targeting therapy using an externally applied magnetic field [13]. With outstanding chemical and physical features and a high surface to volume ratio, new drug delivery systems, incorporating magnetic nanoparticles and having dimensions comparable with those of the body’s macromolecules, are quite appropriate for the successful targeting of cancer cells. Typically, for biomedical applications, one group of commonly used compounds is iron oxide nanoparticles. There are three known natural types, namely magnetite (Fe_3_O_4_), maghemite (γ-Fe_2_O_3_), and hematite (α-Fe_2_O_3_) [14]. Iron oxide nanoparticles are the preferred magnetic nanoparticles due to their hydrophobic features, superparamagnetic properties, low coercivity, and high electric charge, enabling them to act under the organism’s physiological conditions [15]. Iron oxide nanoparticles with different morphologies, coatings, and sizes have been synthesized for biomedical applications [16,17,18]; however, certain properties, such as their bio-distribution patterns, permeability or retention, interactions with plasma proteins, and absorption, could affect the delivery of related drugs to tumors. Nanoparticles based on iron oxides can be stabilized with various organic molecules for different uses in technical or biomedical applications. The biomedical applications of iron-oxide-based nanoparticles require adequate surface properties to ensure interactions with organic structures; these are strongly related to the coating layer properties, especially their non-toxicity and biocompatibility [19]. By combining small sizes, a narrow size distribution, superparamagnetic behavior, low toxicity, and cost-effectiveness, the best magnetic nanoparticles can be designed, which can be used as drug carriers in cancer therapy. There are numerous experimenters that prefer to work with the synthesis method of ferric and ferrous oxide co-precipitation, which is both versatile and available, offering particle property control. The ideal candidate for biomedical applications seems to be iron oxide nanoparticles with a mean physical diameter between 5 and 20 nm, which show the highest cellular uptake and best retention [20,21]. In general, the iron oxide nanoparticles used in biomedical applications should be easily dispersible in an aqueous solution, colloidally stable, and non-cytotoxic in the given concentration range.

Different organic ligands, such as dextran, polyethylene glycol, amino-silanes, and so on, have been shown to be adequate coating shells in the preparation of stable suspensions of magnetic nanoparticles [22,23,24]. Hyaluronic acid (HA) is known as a natural hydrophilic polysaccharide with features of biocompatibility, biodegradability, non-toxicity, an enzyme degradation capacity, and an active tumor-targeting capacity [25]. It interacts with water molecules through hydrogen bonds, and it can act on malignant cells through ligand–receptor interactions [26]. Moreover, HA has attracted the interest of researchers focused on targeted drug delivery to tumors due to its capacity to specifically interact with the CD44 receptor, which is the most expressed receptor on cancer cells [27].

HA can be extracted from various living organisms, either animals or microorganisms, but not plants, using specific procedures of isolation and purification, requiring particularly careful manipulation to maintain the intrinsic properties of polysaccharidic components [28]. The most widespread procedures to obtain HA with different molecular weights use bacterial sources of microorganisms, mainly *Streptococcus zooepidermicus* from marine by-product media or cheese-whey-formulated media [29,30], but also engineered strains of *Bacillus subtilis* [31], *Lacotococcus lactis* [32], and *Escherichia coli* [33]. Some researchers have described HA extraction and purification through the processing of marine biomass sources [34], including some animal waste and by-products, with a focus on fish eyeballs, specifically the vitreous humor of the fish eye [35].

As for the use of HA for magnetic nanoparticle surface modification, we can mention some literature reports. Canava et al. [36] prepared interesting nanoparticles of chitosan/hyaluronan for targeted drug delivery in cancer therapy and emphasized, by in vitro experiments, their fast internalization in human leukemia cells. Dakdouki et al. [37] used HA to modify the magnetic nanoparticle surface for the further targeting of human ovarian cancer cells, due to hyaluronic acid’s affinity for the membrane CD44 receptor. Khodayari et al. [38] reported new results in the domain of drug magnetic carriers for oncologic-targeted treatment. They produced a new nanocarrier based on Fe_3_O_4_ nanoparticles, hyaluronic acid, and β-cyclodextrin for doxorubicin delivery to tumor tissue in the framework of in vitro experiments. The nanocarrier functioned successfully as a pH-responsive system for doxorubicin’s controlled delivery. Zhang et al. [39] reported the preparation of Fe_3_O_4_ nanoparticles stabilized with hyaluronic acid, which were used as a contrast agent in a magnetic resonance imaging study carried out in in vitro experiments. The results of a T2-weighted MRI study performed on HeLa cancer cells were consistent with a lower-intensity signal in comparison to the control cells. The increasing iron concentration led to a more diminished MRI signal. Pramanik et al. [40] yielded some nanocomposite hyaluronic acid–graphene oxide–iron oxide nanoparticles that were loaded with an anticancer drug (doxorubicin) and used in a magnetic hyperthermia experiment. They were tested against cancer cells presenting the surface adhesion receptor CD44. The most promising results recorded in terms of malignant cell destruction were related to the magnetothermal effect exerted by the magnetic nanoparticles. According to the literature, HA-functionalized Fe_3_O_4_ nanoparticles for biomedical uses can be prepared by the hydrothermal method [39,41] or by the co-precipitation method [42]. In other study, the co-precipitation method was applied to obtain magnetic nanocomposites consisting of HA-functionalized Fe_3_O_4_@CeO_2_ nanoparticles [43].

In the study presented here, we applied the co-precipitation method to yield magnetic nanoparticles, coated with hyaluronic acid (HA-MNPs), for future practical applications. Various techniques were used to characterize the formed HA-MNPs, emphasizing the properties that could make them suitable for biomedical uses.

## 2. Materials and Methods

### 2.1. Mathematical Simulation

A theoretical study of hyaluronic acid’s structure was carried out to assess its capacity for interaction with magnetite nanoparticle cations and the surrounding dipolar molecules in water. The mathematical modeling of hyaluronic acid’s optimized structure was accomplished with the PM3 semi-empirical method [44], implemented in the Spartan 18 software package [45], considering a total charge equal to 0 and a number of unpaired electrons equal to 3.

### 2.2. Reagents

Ferric chloride hexahydrate (FeCl_3_·6H_2_O), ferrous chloride tetrahydrate (FeCl_2_·4H_2_O), 25% ammonium hydroxide (NH_4_OH), and hyaluronic acid were acquired from Merck as analytical-grade reagents, used with no supplementary purification.

### 2.3. Preparation of HA-MNP Aqueous Suspensions

**Figure 1:**
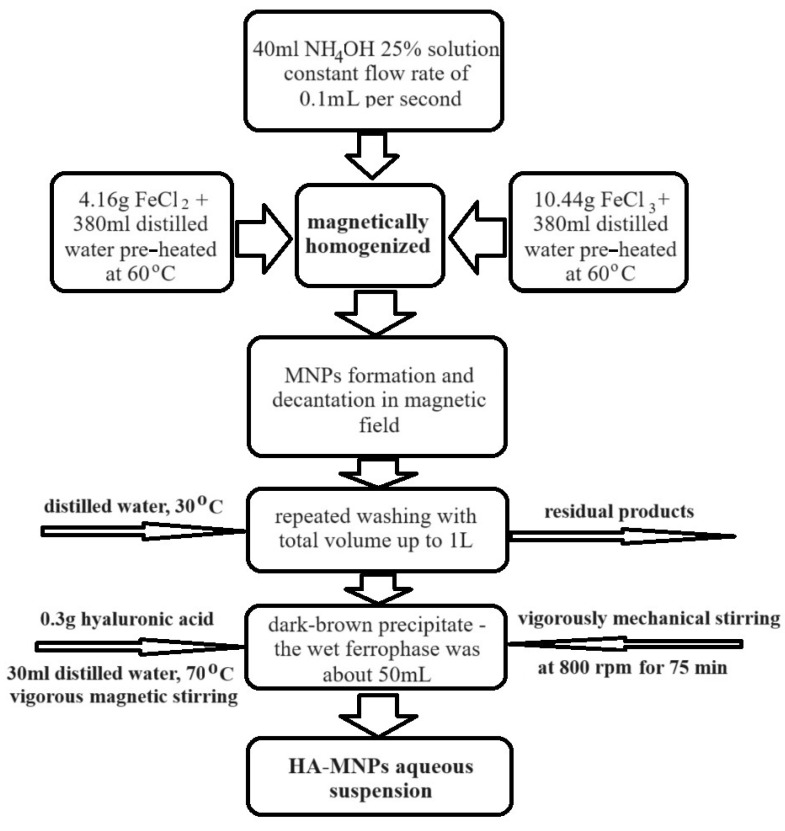
Schematic presentation of HA-MNP synthesis technology.

The magnetic nanoparticles, namely HA-MNPs, were synthesized via an adapted approach involving the controlled chemical co-precipitation of ferric and ferrous iron ions (Figure 1), by using ammonia as an alkaline agent, according to the synthesis protocol de-scribed in [46]. The ferrophase synthesis was conducted at about 60 °C, with subsequent stabilization in a colloidal suspension with hyaluronic acid (C_16_H_27_N_2_O_11_) through electrostatic interactions.

Two solutions of ferric and ferrous chlorides in water were prepared at a relatively high temperature of 60 °C: the first solution was prepared with 4.16 g of FeCl_2_·4H_2_O and 380 mL of distilled water, while the second solution was prepared with 10.44 g FeCl_3_·6H_2_O and 380 mL of distilled water. The precursor iron salt solutions were magnetically stirred at 60 °C and then 40 mL of 25% NH_4_OH was gradually added into the reaction vessel (0.1 mL/s) to trigger ferrophase precipitation [47,48]. The resulting synthesis product was separated at the bottom of the reaction vessel by using a permanent magnet. After removing the supernatant, the ferrophase was washed with about 1 L of distilled water (at about 30 °C) to remove the residual products yielded during the synthesis (like iron hydroxides or certain aliquots of unreacted precursors). The final dark brown product was consistent with ~50 mL of the wet ferrophase, and it was combined with 0.3 g HA dissolved in 30 mL distilled water and then vigorously mechanically stirred for 75 min at 800 rpm. The final aqueous suspension of HA-MNPs had a concentration of ~12.5 mg/mL.

### 2.4. Characterization of HA-MNPs

The TEM images illustrated the size and morphology of the nanoparticles from the diluted suspensions left to dry on 400-mesh copper grids coated with carbon. The TEM device was a Hitachi HD 2700 CFEG STEM model (Hitachi, Tokyo, Japan) that was adjusted to work at 200 kV with electron imaging capabilities. Additionally, the chemical composition of the HA-MNP samples were analyzed using EDS.

The crystalline features of the HA-MNP samples were investigated by analyzing the diffraction patterns provided by a Shimadzu 600 XRD device (0.15418 nm Cu–Kα radiation, Shimadzu, Kyoto, Japan) working in the Bragg–Brentano arrangement between 20 and 80 degrees. Based on the XRD patterns, we calculated the average sizes of the ferrophase crystallite (*D_hkl_*) of the HA-MNPs with the Debye–Scherrer formula [49]:

(1) $$D_{hkl}=\frac{0.9\cdot \lambda }{\beta \cdot cos\theta }\;,$$

*λ* signifies the X-ray wavelength, *θ* is the diffraction Bragg angle, and *β* is the (*hkl*) peak full width at half maximum (FWHM).

The lattice parameter, *a*, can be estimated with relation (2) [50], with *h*, *k*, *l* being the Miller indexes of certain diffraction peaks (*hkl*)—usually the highest one, (311). d*_hkl_* is the corresponding interplanar distance,

(2) $$\frac{1}{d_{hkl}^{2}}=\frac{h^{2}+k^{2}+l^{2}}{a^{2}},$$

and the interplanar distance is

(3) $$d_{hkl}=\frac{\lambda }{2\cdot sin\theta_{hkl}}\;.$$

The magnetic properties study was carried out by applying the vibrating sample magnetometry (VSM) method at 22 °C or room temperature and using the MicroMag Model 2900/3900 magnetometer (Lake Shore Cryotronics, Inc., Westerville, OH, USA), which provided the magnetization curve, the value of the specific magnetization at saturation, and the value of the coercive magnetic field of the HA-MNPs. Based on the magnetization data, and assuming a spherical shape for the nanoparticles, we could estimate the average value *d_M_* of the magnetic core size, at the environmental temperature *T*, using Langevin’s equation [51]:

(4) $$d_{M}^{3}=\frac{18\cdot K_{B}\cdot T}{\pi \cdot \mu_{0}\cdot M_{S}\cdot m_{S}}{\left(\frac{dM}{dH}\right)}_{H\to 0},$$

*K_B_* is Boltzmann’s constant, *M* and *m_S_* represent the specific magnetization saturation of the analyzed sample and that of the bulk magnetite (0.48 × 10^6^ A/m) [52], respectively, and *μ*_0_ is the vacuum’s magnetic permeability.

We recorded the vibration spectrum using the ATR-FTIR technique with an Alpha device from Brucker (Karlsruhe, Germany), via a non-destructive method using a ZnSe crystal. The recording resolution was 4 cm^−1^ at room temperature (22 °C), between the wavenumbers of 600 and 4000 cm^−1^.

To examine the distribution of the colloidal particles’ diameters, i.e., the hydrodynamic diameter, as well as the particle concentration, nanoparticle tracking analysis (NTA) was applied. We worked with a NanoSight LM20 device (NanoSight Ltd., Wiltshire, UK) equipped with a CCD camera for high-speed video capture (operated at 30 frames per second) and using a red laser at 22 °C or room temperature and a 10^−4^ volume dilution of the HA-MNP sample. Dedicated nanoparticle tracking analysis software (NTA v3.2) and finite track length analysis (FTLA) were used for size determination.

The DSC and TG investigations were carried out by scanning the HA-MNPs in a nitrogen atmosphere at a 50 mL/min flow rate, between 25 °C and 850 °C and at a 10 °C/min heating rate, with a sample weight of 15 mg, using the SDT Q600 calorimeter (TA Instruments, New Castle, DE, USA). The results of the DSC analysis (heat flow variation) were presented in W/g, while the results of the TG analysis (weight loss) were given as percentages (with the Universal Analysis 2000 software).

### 2.5. Nanotoxicity Test

A preliminary experiment was carried out to test the HA-MNPs’ nanotoxicity towards environmental vegetation. Twenty intact corn caryopses (for treated samples and a control, non-treated one), identical in size and color and free from wrinkles, were left to germinate in plates on moistened filter paper in a laboratory room at the Sibiu University laboratory, in controlled conditions at 24 ± 0.5 °C and complete darkness. In the test plate, 15 mL of an aqueous solution of HA-MNPs, with a 50 µL/L volume fraction, was added, while the control sample was left to germinate in the same environmental conditions, using 15 mL of distilled water. Two–three-day-old germinated caryopses, having a 1–2 cm root length, were selected for cytogenetic analysis. The root tips were fixed in Carnoy solution (1 glacial acetic acid/3 absolute ethanol; *v*/*v*) for 24 h and stored in 70% ethanol at 4 °C in a refrigerator. For staining, the root tips were softened in 37% HCl:distilled water solution (1:1) for 25 min, and then maintained in modified carbol-fuchsin dye [53] in a refrigerator. For each variant, we prepared five microscope slides via the squash technique [54], using individual root tips from each germinated caryopsis, which were crushed on the slide, and poured one drop of 45% acetic acid [55]. For each slide, at least 2000 cells and over thirty microscopic fields per slide were analyzed by the same operator using a Euromex IS 1153-EPL microscope (40× objective, Euromex Optics, Arnhem, The Netherlands). The relevant abnormal cell photos were taken with the CMEX-18000-PRO digital camera and Euromex ImageFocus Alpha software (vers. x64).

## 3. Results

**Figure 2:**
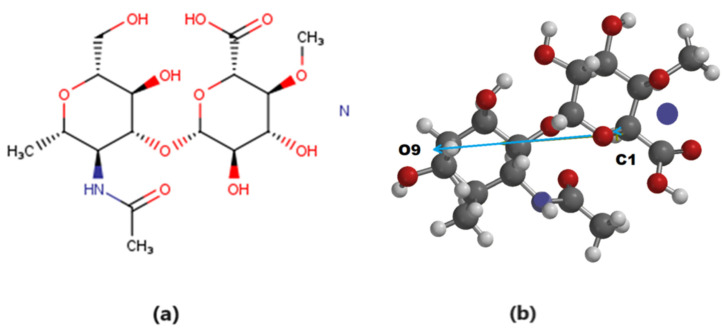
(**a**) Hyaluronic acid molecule (C_16_H_27_O_11_N_2_) [56]; (**b**) energetically optimized molecular structure with dipole moment—blue arrow. Oxygen atoms are colored in red, carbon atoms in dark grey, hydrogen in light grey, and nitrogen atoms in dark blue.

The structure of hyaluronic acid (Figure 2a), provided by PM3 algorithm modeling after energy optimization, is given in Figure 2b.

**Figure 3:**
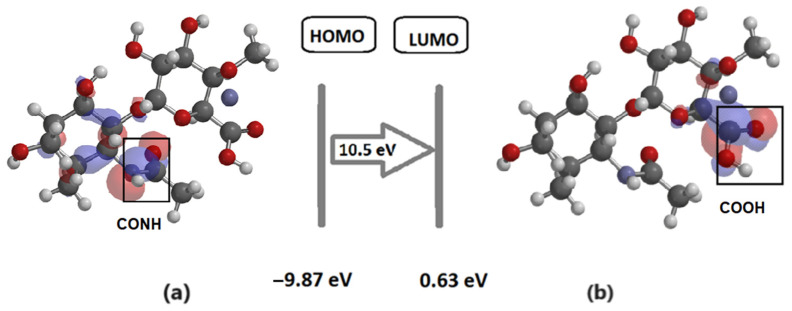
(**a**) Representation of HOMO orbital corresponding to ground state molecule; (**b**) representation of LUMO orbital for the excited state molecule. Red balls represent oxygen atoms; dark grey balls represent carbon atoms; light grey balls correspond to hydrogen atoms. Translucent blue and red areas represent electronic cloud distribution below and above the molecule, respectively.

In Figure 3a,b, the energetically optimized structure of HA (C_16_H_27_O_11_N_2_) is represented, with the frontier electronic orbitals, the highest occupied molecular orbital (HOMO), and the lowest unoccupied molecular orbital (LUMO).

**Figure 4:**
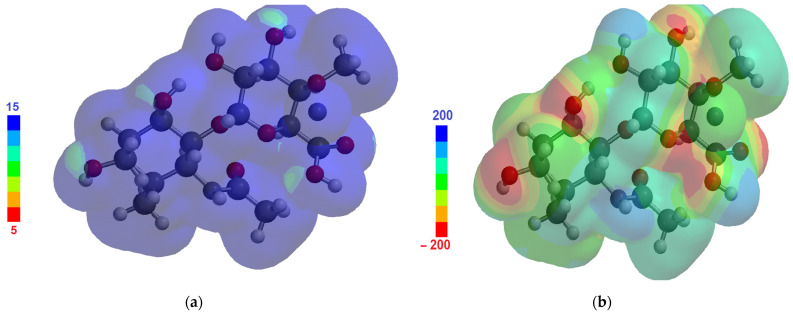
(**a**) Ionizing potential map; (**b**) electrostatic potential map. Numerical values are in eV.

The HOMO–LUMO gap energy was found at 10.5 eV (with HOMO energy of −9.87 eV and LUMO energy of 0.63 eV); the electrostatic map (Figure 4a) and the ionization map (Figure 4b) are represented in Figure 4.

In Figure 4a, the ionizing potential distribution shows values around 10 eV throughout the molecule’s approximate surface, concordant with the energy of the HOMO orbital (9.87 eV). In Figure 4b, the electron surface density is presented, with the colors indicating the values of the electrostatic potential on the molecule surface (the red color corresponds to negative potential and denotes a stabilizing interaction between the molecule and a positive charge, while the blue color corresponds to positive electrostatic potential).

**Figure 5:**
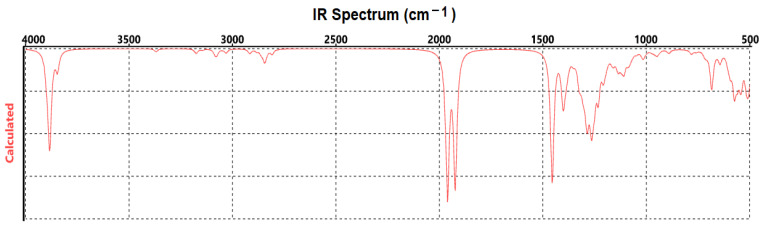
Theoretical vibration spectrum of HA optimized structure.

The theoretical vibration spectrum calculated by PM3 mathematical modeling (Figure 5) showed relatively high-intensity bands at wavenumbers over 3500 cm^−1^, which resulted from the stretching vibrations of O-H and N-H groups; moderate-intensity bands around 2850 cm^−1^, given by C-H stretching vibrations; and C=O and C-O vibrations at about 1800 cm^−1^ (shifted compared to experimental spectra). In the range of 1050–1150 cm^−1^, the vibrations of C-OH bonds, C-O (exocyclic), and C-C and C-O-C groups were obtained, while, at around 600 cm^−1^, the skeleton vibrations were evidenced [57].

**Figure 6:**
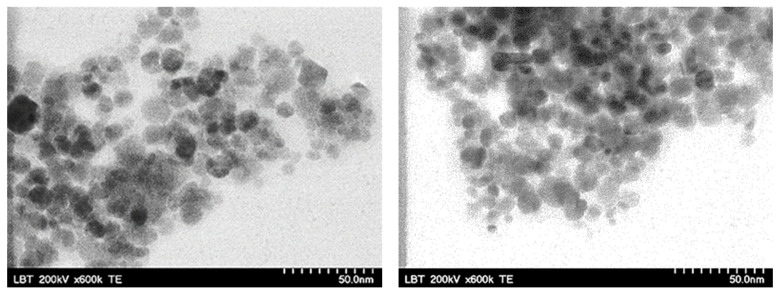
TEM images of HA-MNPs (scale bar = 50 nm).

**Figure 7:**
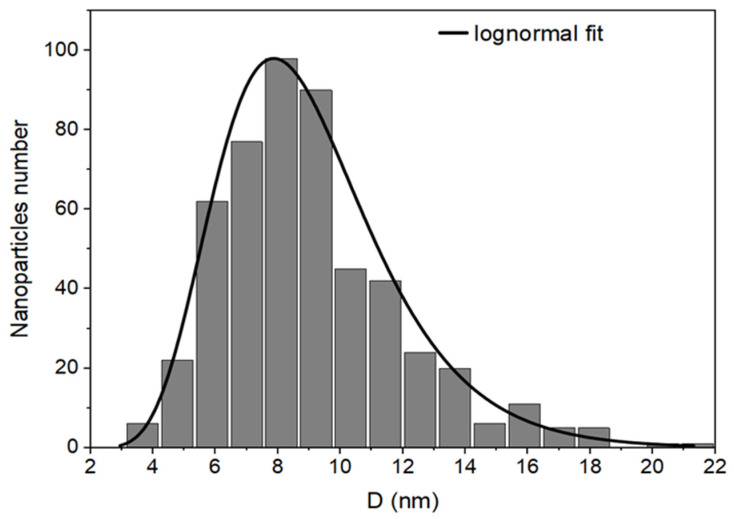
Size distribution histogram of HA-MNPs for 515 particles from 10 different TEM images and lognormal fitting.

The morphology and the physical size of the HA-MNPs were characterized by TEM imaging. The TEM pictures revealed the quasi-spherical shape of the nanoparticles (Figure 6), with a size distribution between 5 and 20 nm (Figure 7), in accordance with other reports (5 to 17 nm) [39] and in contrast to others (15 to 49 nm) that reported larger size histograms [58]. The histogram of HA-MNPs was built with values measured for 515 nanoparticles from 10 different TEM images with the ImageJ software (vers.1.8.0) and was plotted using the OriginLab software (vers. 2023b). The histogram fitting was performed with a lognormal function, which provided the median value and the standard deviation (8.29 mm and 2.33 nm, respectively, with the correlation coefficient being R^2^ = 0.945).

The TEM pictures revealed nanoparticles with a globular shape, with a mean size of 9.05 ± 2.89 nm, mostly between 3 and 20 nm and without visible differences in the nanoparticle morphology. There were no visible particle agglomerations or large particles with eventual ferrimagnetic features; thus, further below, the VSM investigation showed a remarkably small hysteresis loop at the center of the magnetization graph. However, certain rare clusters could exist, as evidenced by the NTA result, consistent with the multi-modal histogram of the hydrodynamic diameter. The small physical size of the magnetic particles, the lack of particle agglomeration, and the resulting stability in an aqueous colloidal suspension make HA-MNPs suitable for use in the biomedical field. We visually observed [59,60] the status of the HA-MNP suspension’s stability by examining the eventual precipitation at the bottom of the bottle. After the first three months, we noticed a slight deposit; thus, we vigorously shook the bottle, and then the suspension appeared to remain stable for two more weeks. No further homogenization was observed in the fifth month, with clear phase separation occurring.

**Figure 8:**
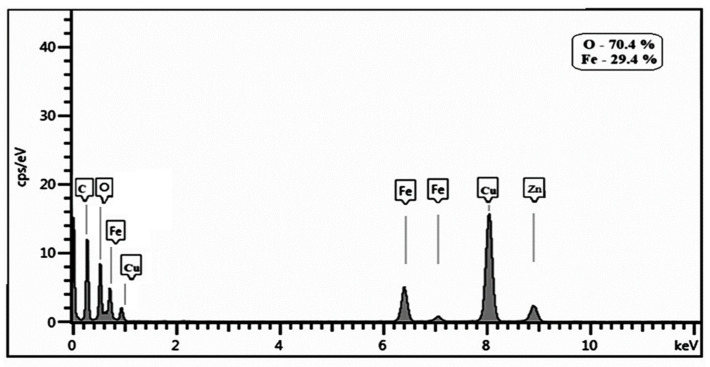
EDS pattern of synthesized HA-MNPs.

The EDS pattern presented in Figure 8, regarding the elemental analysis of the HA-MNPs according to the device’s sensitivity threshold, evidences the sample’s main components, i.e., iron and oxygen, while copper and zinc signals were detected from the sample’s grid support. In addition, the carbon line could indicate the presence of HA but also the carbon film support or carbon contamination from the environment.

**Figure 9:**
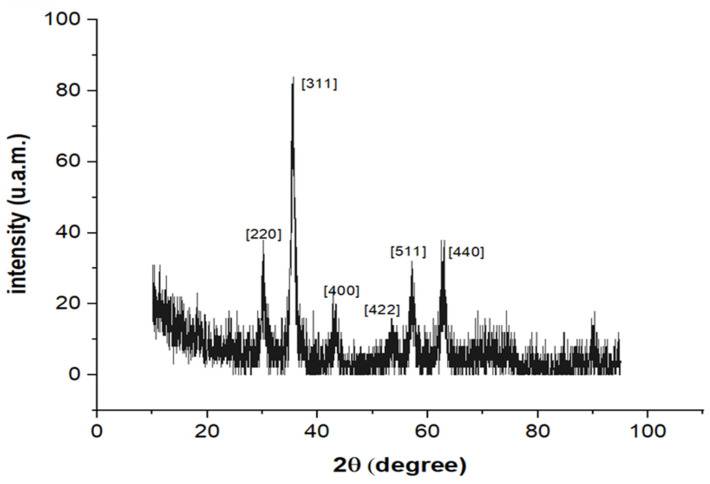
XRD pattern of synthesized HA-MNPs.

The XRD pattern for the HA-MNPs is presented in Figure 9; the positions of all evidenced diffraction peaks, (220), (311), (400), (422), (511), and (440), were in accordance with the cubic phase of Fe_3_O_4_ nanoparticles (JCPDS Card No. 75-0449). Based on the XRD patterns and using Debye–Scherrer’s formula for the highest-intensity peak, namely (311), the mean crystallite size of the HA-MNPs was estimated at about 8.35 nm; this value is concordant with the median value resulting from the TEM investigation. The spherical shape of the nanoparticles was assumed, as suggested by the TEM imaging, since no significant differences in FWHM were observed in the characteristic diffractogram (with sharp peaks of relatively high intensity and no amorphous phase).

Other crystallinity features could be discussed, such as the lattice parameter and the interplanar distance of ferrophase crystallites. The lattice parameter, a, and interplanar spacing, d, were estimated from the XRD patterns using Bragg’s diffraction law [50], based on the mathematical relations given in the Materials and Methods. For the lattice parameter, we obtained a value of 0.8367 nm, which is lower than the value of bulk magnetite (a = 0.839 nm) [61] and higher than the value of bulk maghemite (a = 0.834 nm) [62]. Cuerca et al., studying the magnetite to maghemite transition, revealed that the lattice parameter, a, decreased due to magnetite’s oxidation to maghemite [63]. Thus, the value obtained by us for the studied HA-MNPs could indicate that the magnetite nanoparticles were partially converted to maghemite during the nanoparticle synthesis process. This could occur when an increased temperature is applied during nanoparticle synthesis in the presence of oxygen, thus favoring the oxidation of magnetite to maghemite [64]. Moreover, for the interplanar spacing, we obtained a value of d_311_ = 0.2523 nm, which is close to the value corresponding to bulk magnetite (d_311_ = 0.253 nm) [65].

**Figure 10:**
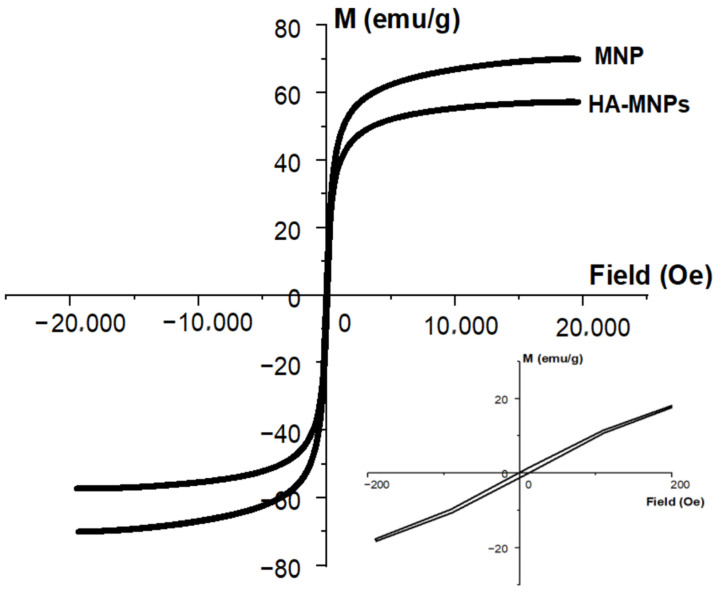
Magnetization curves of synthesized HA-MNPs and uncapped magnetic nanoparticles (MNP). Inset: expanded view of central area of hysteresis loop for synthesized HA-MNPs.

In Figure 10, one can see the curve of sample magnetization versus the external magnetic field as resulted from the VSM investigation, from the analysis of which the value of 57.29 emu/g for the saturation was taken to estimate the magnetic core diameter, as well as the value of the coercive field, which was 6.65 Oe. The magnetization curve emphasized the superparamagnetic features of the HA-MNPs, with saturation behavior and a very small coercive field. The saturation magnetization of the uncoated powder (MNP) was significantly higher (about 69.4 emu/g) than for the HA-MNP product (57.29 emu/g), with the difference being related to the dilution effect of the molecular coating shell.

Following Langevin’s equation (Equation (4)), the magnetic core diameter of the HA-MNPs of about 8.31 nm at 295 K was estimated, which is similar to the median value of the physical diameter of the studied nanoparticles, obtained by the TEM measurements.

**Figure 11:**
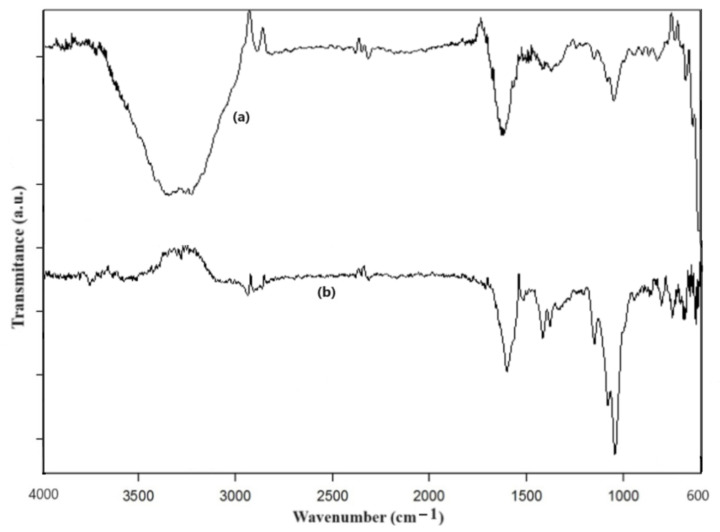
ATR-FTIR spectra of (**a**) synthesized aqueous solution of HA-MNPs and (**b**) 0.02 g/mL solution of HA, recorded with water background.

Analyzing the ATR-FTIR spectrum recorded with a water background (Figure 11), we found the bands of the amide group from the HA molecule. The vibration peak at 1653 cm^−1^ is typical for the asymmetric vibration of carbonyl, which is described in chemistry tables as ranging within the 1690–1630 cm^−1^ frequency interval [66] and found in pure HA molecules at 1599 cm^−1^ [67] or at 1604 cm^−1^ [68]. Lower-intensity vibrations could be observed in the 1500–1250 cm^−1^ range, corresponding also to amide group vibrations [67]. The vibrations at about 1045 cm^−1^ were assigned to the C-OH and C-C groups, while the moderate-intensity bands at around 2880 cm^−1^ could be assigned to C-H stretching vibrations [57]. The amide N-H stretching was evidenced at 3208–3196 cm^−1^, in line with the chemistry tables [62], which give a range of 3700–3500 cm^−1^, and with the amide band in pure HA molecules, at 3300–3500 cm^−1^ [67] or at 3257 cm^−1^ [68].

The results obtained by the ATR-FTIR method are concordant with those reported in [39], where FTIR recordings for a HA-Fe_3_O_4_ sample were presented. The skeleton vibrations of the ferrophase were found as expected at frequencies lower than 600 cm^−1^, and the ATR-FTIR device that we used allowed the observation of a relatively large band at this frequency. This IR band is representative of the stretching vibration of Fe-O bonds in the crystalline lattices of iron oxide nanoparticles (magnetite and maghemite) [69,70,71], being also overlapped with the HA skeleton’s vibration bands. Other researchers obtained similar results [72,73]. The HA binding to the ferrophase nanoparticles resulted in a wavenumber shift for the C=O vibration from 1653 cm^−1^ to 1600 cm^−1^ and the band intensity decreased for the 3200 cm^−1^ peak (stretching vibrations of O-H and N-H groups) and the 1045 cm^−1^ peak (C-OH and C-C groups).

**Figure 12:**
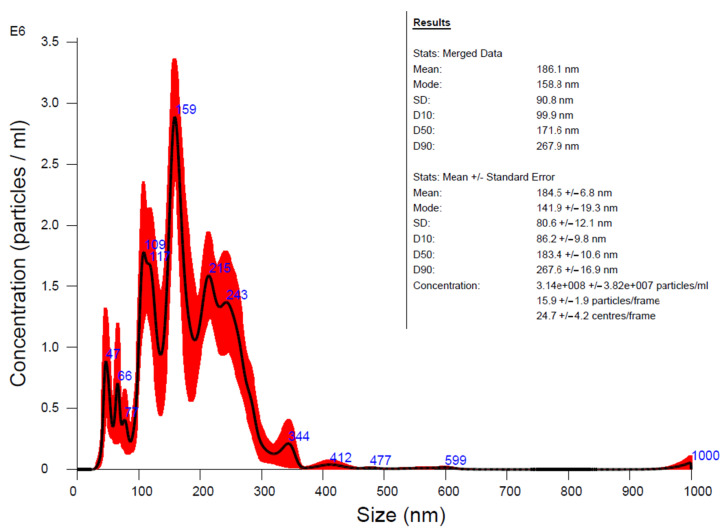
Size distribution estimation from NTA measurements for 10^−4^ volume diluted HA-MNPs—averaged finite track length analysis (FTLA) concentration/size graph for experiment. Inset: detailed results of NTA measurements for HA-MNP sample.

The hydrodynamic diameter of colloidal nanoparticles is generally larger than their physical diameter, as provided by microscopy techniques, since, during thermal movement within the dispersion fluid, each particle is surrounded by a fluid volume containing coating molecules together with dispersion medium molecules. The intermolecular forces developed between the nanoparticle coating shell and the dispersion medium could result in clusters of different sizes, which could display a different size distribution compared to that resulting from microscopy methods. Moreover, the fluid clusters could bring together a pair of nanoparticles in the same matrix envelope; thus, several distinct nanosystem sub-populations could appear in the analysis result. This seems to be also the case in our data, with a multi-modal hydrodynamic diameter size distribution and the maximal appearance frequency for about 159 nm particles (Figure 12). Secondary peaks at over 200 nm could be assigned to rare aggregates, which could have been caused by the interactions among the nanoparticles in the solution, as also observed by others in similar systems [39,41]. This is concordant with [39], where magnetic nanoparticles coated with hyaluronic acid were reported to have a hydrodynamic diameter between 270 and 310 nm. A particle concentration for HA-MNPs of about 3.14 × 10^12^ particles/mL was detected, according to the analysis results presented in Figure 12.

**Figure 13:**
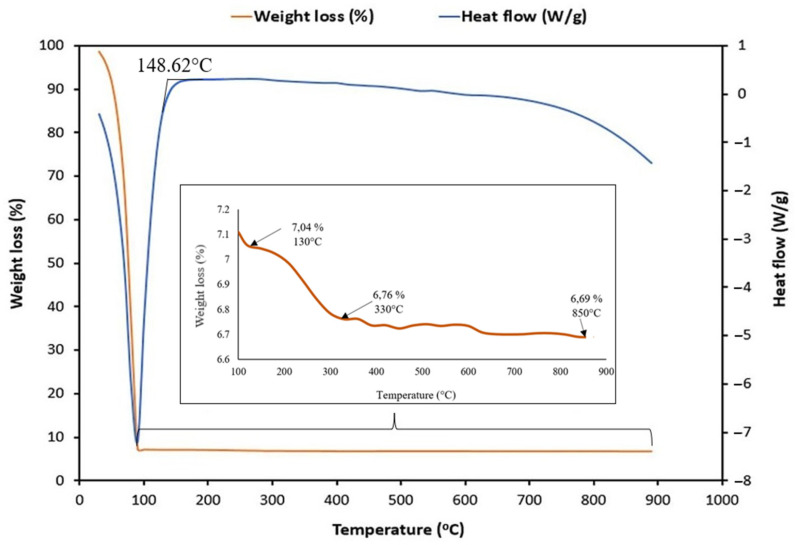
Weight loss (%) and heat flow (W/g) of HA-MNPs. Inset: expanded view of weight loss for higher temperatures than 100 °C.

The weight loss (%) and heat flow (W/g) of the HA-MNP sample are presented in Figure 13. Two distinctive weight losses were observed. In the first stage, at temperatures below 130 °C, a remarkable weight loss was observed, with up to only 7.04% of the initial weight remaining at 130 °C. This loss can be explained mainly by the evaporation of water from the sample, with a certain contribution from the degradation of HA. Mondek et al. [74] showed that the degradation of HA is significant in the range of 90–120 °C. The second stage of sample transformation, recorded in the temperature range between 130 and 330 °C, when the whole sample weight diminished from 7.04% to 6.76%, could be assigned to HA degradation. This is concordant with Ahire et al. [75], who found a weight reduction for HA between 180 and 330 °C, which was attributed to the degradation of the polysaccharide part. In addition, Larrañeta et al. [76] obtained, for pure HA, a maximum value in the exothermic process at 240 °C and attributed this phenomenon also to the degradation of the polysaccharide part. Atrei et al. [77] reported similar temperature ranges at which degradation occurred. Next, from 330 up to 850 °C, there was no significant decrease in mass (from 6.76% up to 6.69%), indicating the stability of the nanoparticles at higher temperatures, in line with Hasan et al. [78], who detected no significant weight loss after 575 °C.

**Figure 14:**
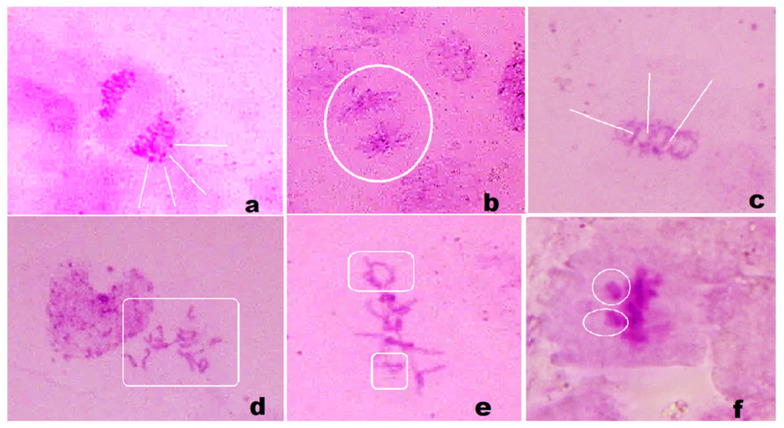
Examples of abnormal cells found in root tips of HA-MNP-treated corn caryopses: (**a**) sticky anaphase with micronucleus; (**b**) star anaphase; (**c**) anaphase with interchromatin bridges; (**d**) C-metaphase; (**e**) laggard and ring chromosomes at metaphase; (**f**) sticky metaphase with chromosome fragments. The white lines, circles, and rectangles indicate the specific abnormality in the dividing cells.

Images of some abnormally dividing cells observed during this preliminary microscopic analysis are presented in Figure 14 as follows: (a) sticky anaphase with micronucleus; (b) star anaphase; (c) anaphase with interchromatin bridges; (d) C-metaphase; (e) laggard and ring chromosomes at metaphase; (f) sticky metaphase with chromosome fragments. These first results indicate that the HA-MNPs’ internalization during the germination process was able to influence the mitotic division in the root tip cells of the corn caryopses, mainly the anaphase and metaphase of mitosis. We found that the HA-MNP nanoparticles, synthesized by us, could induce chromosomal aberrations in the radicular meristem cells. 

## 4. Discussion

The study carried out by us was focused on the production of magnetic nanoparticles that can be useful in magnetically assisted pharmaceutical delivery. HA was chosen to stabilize the nanoparticles in water, due to its biocompatibility and non-toxic properties, its active tumor-targeting capacity, and it capacity to develop bonds both with the nanoparticle surface and with some drugs that are loaded onto nanoparticles.

Analyzing the electronic cloud of the HA optimized structure, we could see that, in the molecule’s ground state (Figure 3a), the electronic density appeared to be localized mostly on the amide group (CONH), while, in the excited state, it was the carboxylic group (COOH) in proximity to the C1 carbon atom that attracted the electronic cloud (Figure 3b). The electric dipole moment was found to be 3.44 D (Figure 2b), oriented from the proximity of the C1 carbon atom (+1.09 e charge) towards the negatively charged oxygen O9 (−0.563 e), as resulted from the electrostatic charge calculation.

Considering HA’s capacity for interaction with the magnetite ferrophase, the side atom groups of negatively charged HA, represented by reddish spots in the electrostatic map in Figure 4a (corresponding to oxygen atoms in the vicinity), were able to interact with cations on the magnetic particle surface. The relatively high dipole moment (3.44 D) compared to that of water (1.84 D [79]), also suggests that electric interactions could successfully develop in the HA-MNP system and with the surrounding water molecules. Since the molecular properties of HA underlie its good interaction capacity with ferrophase cations and surrounding water molecules, it is expected to enable the reliable capping of the nanoparticle surface and consequently good stability in water dispersions, better than for other core–shell magnetic nanosystems. The electric dipole moment, which was found to be 3.44 D, is comparable with that of citric acid, which is 2.47 ± 1.08 D [80], known as a good stabilizing coating molecule for magnetic nanoparticles. The HA dipole moment is higher than that of perchloric acid, which is 2.17 D [81]; gallic acid, which is 2.41 D [82,83]; and aspartic acid (1.6 D) [84]. These molecules were all used to obtain stable dispersions of magnetic nanoparticles; thus, we found hyaluronic acid to be a very promising stabilizer for the coating of ferrophase particles.

The vibration bands recorded experimentally were close to the theoretically modeled ones. For the C-OH group and C-C groups, we recorded the peak at 1045 cm^−1^, compared to the theoretical one at 1080 cm^−1^. For the C-H stretching, we recorded the band at 2880 cm^−1^, while, in the simulated spectrum, there was a band at 2850 cm^−1^. Some shifts were noticed to lower wavenumbers, such as for the stretching vibrations of the O-H and N-H groups (around 3200 cm^−1^, compared to over 3500 cm^−1^ in the theoretical spectrum) and carbonyl stretching (1653 cm^−1^, compared to about 1800 cm^−1^ in the theoretical modeling). One could say that the experimentally recorded vibration bands, in line with the literature reports on the spectral analysis of HA, provide proof of HA’s interaction with the magnetite nanoparticle’s surface.

We followed the careful application of chemical co-precipitation to obtain a ferrophase with the best dimensionality and magnetizability properties that was coated with HA. The results presented in the previous section for the successfully prepared sample demonstrate this. The physical dimensions of the magnetic nanoparticles were small enough, compared to those of other authors [41,42,43], and the nanoparticles had better saturation magnetization than other studies have reported [73]. The HA shell binding proceeded successfully, as evidenced by the nanoparticles’ stable dispersion in water despite the destabilizing magnetic dipole–dipole attractions, with the binding confirmed by the ATR-FTIR analysis. The behavior regarding the variation in the external magnetic field was manifested by superparamagnetism. This is required in applications such as drug targeting, which involves the reversibility of the total magnetic moment, i.e., of the specific magnetization, with a remarkably small remanence, as shown by the coercive field evidenced by VSM. The behavior of the HA-MNPs in a fluid medium was tested by NTA on a dilute suspension, with the results being comparable to those of other reports in the literature [39].

Our sample, with a crystallite size of about 8.3 nm and a nanoparticle diameter of 9 nm, had similar values when compared to other samples of magnetic nanoparticles coated with HA [42,43], also prepared by the co-precipitation method.

Soleymani et al. reported approximately 8.5 nm magnetic nanoparticles coated with HA [73] prepared by the hydrothermal method. For other HA-coated magnetic nanoparticles, prepared by hydrothermal synthesis, higher physical particle mean diameters were estimated, like 10.2 nm [39], 15.6 nm [41], 13 nm [42], and 15 nm [43]. As is known, a small diameter favors nanoparticles’ endocytosis in the cells [85]. Magnetic nanoparticles loaded with pharmaceuticals do not grow significantly larger. This is because the attached molecules have dimensions that are several orders of magnitude lower than the nanoparticles. Thus, a small size ensured for the magnetic carriers from the synthesis procedure could guarantee the targeting of tumoral cells. Moreover, the smaller the nanoparticles, the larger the surface to volume ratio, which increases the efficacy of interacting with environmental fluids.

The hydrodynamic diameter size distribution is multi-modal and presents a maximal appearance frequency at about 159 nm (Figure 12). Subpopulations of smaller-sized colloidal particles are expected to show lower-intensity peaks at about 109 and 66 nm, while secondary peaks at over 200 nm are the result of colloidal particle association in solution, as in other similar systems [39,41]. Indeed, Lee et al. [43] concluded that there is a clear tendency towards particle agglomeration in aqueous suspensions; thus, additional modification of the nanoparticle surface is needed to improve the dispersibility. This is concordant with [39], where magnetic nanoparticles coated with hyaluronic acid were reported to have hydrodynamic diameters between 270 and 310 nm. It was shown that a temperature increase from about 23 to 35 °C diminished the hydrodynamic diameter up to two times [86]; thus, when delivered in blood at physiological temperatures (36 to 37 °C), the actual hydrodynamic diameter becomes smaller than that measured at the environmental temperature (about 22 °C). In addition, in the blood, macromolecules like proteins tend to surround the nanoparticles, increasing their hydrodynamic diameter, as reported by Yeremenko et al. [87], who found a 10 nm increase in mice serum.

The XRD analysis results for our sample confirmed the formation of crystalline iron oxide nanoparticles, being similar to the results reported by other researchers [39,43]. As no additional diffraction peaks were detected, one could assume that neither intermediate reaction products nor undesired impurities in the co-precipitation reaction remained in the sample. Any impurity could hinder the process of molecular shell binding and consequently the stability of the aqueous suspension of magnetic nanoparticles, but this did not happen in our case, with the HA binding being confirmed by ATR-FTIR.

Compared to other HA-coated magnetic nanoparticles described in the literature, like those prepared by Fang et al. [88], with 41 emu/g saturation magnetization, our sample emphasized better magnetizability with relatively high saturation magnetization (57 emu/g) and a very small coercive field (about 6.6 Oe), which correspond to superparamagnetic behavior. Soleymani et al. reported that the saturation magnetization increased from 11.3 to 29.6 emu/g for iron oxide nanoparticles coated with HA after hydrothermal treatment [73]. The magnetization properties are necessary for biomedical use in magnetically assisted drug delivery, when external magnetic fields are applied for magnetic nanocomposite use. For the successful operation of magnetically assisted drug delivery, high magnetization is needed for the controllable manipulation of the magnetic nanosystem in the human body’s structures, while superparamagnetism, ensured by a very small coercive field, is necessary to avoid remanent magnetization to the external magnetic field’s removal—avoiding any size effects related to the magnetic field’s influence on the cellular structures. A small size confers these magnetizability properties and prevents particle agglomeration during circulation through the patient’s body, as well as in the targeted organs [89,90,91].

The investigation with the ATR-FTIR technique evidenced HA’s interaction with the nanoparticle surface, since the main vibration bands were present in the recorded spectrum, being very similar to those reported in other studies [72]. Consequently, the applied procedure provides core–shell nanoparticles suitable for the further coupling of pharmaceutical products needed in oncologic chemotherapy. This was demonstrated by Zhou et al. [42], Zhang et al. [39], and El-Dakdouki et al. [37], who successfully used HA-coated magnetite nanoparticles for in vivo tests, and also by our results obtained through ATR-FTIR. The stabilizing action of the HA coating shell was consistent with the uniformly dispersed magnetic nanoparticles, which did not precipitate from the sample, in contrast to the uncoated ferrophase.

The identification of chromosomal aberrations was performed based on the literature [92,93,94,95] and our previous experience [96,97]. The underlying mechanisms of HA-MNPs’ interaction with the cellular nucleus involve the suspension’s uptake at the level of rootlets, diffusion towards the cell cytoplasm, iron cations’ release from the nanoparticle surface, and their penetration into the cell nucleus, where a catalytic effect is supposed to occur. Thus, water molecules could be split by Fenton reactions mediated by iron ions with the release of free water radicals. These radicals induce a toxic effect, impairing chromosome evolution during mitosis. In this way, abnormal cell divisions could be initiated. Some of them could be compensated for by the defense mechanisms of cells, but others are expected to be perpetuated as genetic mutations. In the next study, we will develop an experiment by varying the HA-MNP suspension’s dilution and by assessing quantitatively their effects on the dividing cells, in every phase of mitosis. These results will contribute towards improving the research field of magnetic nanoparticle synthesis with well-addressed biomedical uses. Due to its properties, this magnetic nanoparticle sample appears as a promising tool for the loading of pharmaceuticals and their safe delivery to the affected organs.

## 5. Conclusions

In summary, we synthesized a colloidal suspension of magnetic nanoparticles coated with HA via the co-precipitation method, for further biomedical applications. Our HA-MNP sample, dispersed in water, was synthesized by an available and facile co-precipitation method at a low cost. The TEM analysis showed that the core–shell structure of the HA-MNPs had a globular shape with a small mean size of around 9 nm, although relatively large colloidal particle associations were formed in the dispersion fluid, as suggested by the hydrodynamic diameter distribution. The XRD analysis evidenced the crystallinity properties of the HA-MNP sample, with an average crystallite size of 8.35 nm and no amorphous phase. The EDS analysis of the elemental compositions of our sample denoted the presence of iron and oxygen. The investigation of the HA-MNP product with the ATR-FTIR technique revealed the interaction of HA and the ferrophase particle surface. The superparamagnetic behavior of the HA-MNPs was revealed by the VSM analysis results, with our sample having a saturation value of magnetization of about 57.29 emu/g and low magnetic coercivity of about 6.65 Oe. Due to its properties, this magnetic nanoparticle sample is recommendable for use in the biomedical field. Although further biomedical studies are needed, our results, compared with the other studies mentioned in this work, indicate that HA-MNPs represent an attractive nanosystem that could exhibit great potential for biomedical uses. We plan to test the HA-MNPs’ biocompatibility on healthy and malignant mammalian cell cultures, while its nanotoxicity in young plantlets was evidenced by the aberrant mitoses induced in corn plantlets.

## Data Availability

All data are contained within the article.
